# Evaluation of the Effect of Organic Matter from Invasive Plants on Soil Nematode Communities

**DOI:** 10.3390/plants12193459

**Published:** 2023-09-30

**Authors:** Michaela Jakubcsiková, Lenka Demková, Marek Renčo, Andrea Čerevková

**Affiliations:** 1 Institute of Parasitology, Slovak Academy of Science, 040 01 Košice, Slovakia; jakubcsikova@saske.sk (M.J.); renco@saske.sk (M.R.); 2 Department of Ecology, Faculty of Humanities and Natural Sciences, University of Prešov, 080 01 Prešov, Slovakia; lenka.demkova@unipo.sk

**Keywords:** soil nematodes, *Fallopia japonica*, *Solidago gigantea* invasion, organic matter, soil properties, pot experiment

## Abstract

Invasive plants can cause loss of biodiversity and degradation of ecosystems with varying degrees of impact on soil communities. Little is known about how the organic matter of these invaders in the soil affects soil properties and nematode communities. We performed a pot experiment with non-invaded grassland soil and organic matter from two invasive plants, *Fallopia japonica* and *Solidago gigantea*, to assess and compare the composition and function of the nematode communities and soil properties. We tested five treatments: (1) non-invaded grassland soil (S), (2) 100% decayed organic matter from *F. japonica* (OMF), (3) 100% decayed organic matter from *S. gigantea* (OMS), (4) 50% soil plus 50% organic matter from *F. japonica* (S/OMF), and (5) 50% soil plus 50% organic matter from *S. gigantea* (S/OMS). Analysis of nematode composition was conducted over five months from May to September. The number of identified genera and diversity index was highest in the S treatment. The soil moisture content was highest, pH and the diversity index were lowest and herbivorous nematodes were absent in OMF and OMS treatments. The addition of OMF and OMS to soil decreased the soil pH and moisture content and increased the contents of organic carbon and total nitrogen. In S/OMF, the abundance of herbivores was lower than in S and the abundances of bacterivores and fungivores decreased during the study period. In the S/OMS, a significantly high diversity index was observed, similar to that in the S treatment. The selected ecological and functional indices differed between S/OMF, S/OMS and S, but not significantly. Our findings indicated that the organic matter from the two invasive plants could differentially contribute to interactions with nematode communities. A decrease in productivity and the slowing of nutrient cycling demonstrated by the decrease in the abundances of bacterivores and fungivorous nematodes may be common adding organic matter of invasive plants to soil. A decrease in the abundance of herbivores after the application of organic matter of *F. japonica* could potentially be used as an ecologically friendly management strategy against plant parasitic nematodes.

## 1. Introduction

The presence of nonindigenous invasive plant species within ecosystems has been associated with negative impacts on global biodiversity, ecosystem processes, ecosystem services, economic systems, and human well-being [1,2]. Plant invaders can directly modify the soil environment by releasing root exudates that affect soil structure, mobilise and/or chelate nutrients, and alter soil food webs differently from native species [3]. Additionally, plant invaders often produce substantially larger amounts of easily decomposed litter that turns over faster than in the plant communities they replace [4,5]. The decomposed organic matter from invasive plants may have different effects than those exerted by the invasive plants themselves on the surrounding environment during their growth season [6]. For example, Wakjira et al. (2009) [7] compared the impacts of compost and fresh biomass from the invasive *Parthenium hysterophorus* L. and found that compost from *P. hysterophorus* had a significantly lower allelopathic effect on the germination and growth of native lettuce and was less toxic compared to fresh plants. Jiao et al. (2021) [8] recorded a similar positive effect of composted invasive crofton weeds (*Ageratina adenophora* (Spreng.) R.M.King & H.Rob.), whose organic matter contains much fewer allelopathic compounds as living plants and greatly increases nutrient content, the growth of ryegrass, enzymatic activity and microbial diversity. However, Das et al. (2018) [9] reported contradictory results in a study on the effect of the aqueous extract and organic matter from *A. adenophora* on some winter crops and weeds. High concentrations of an aqueous extract and compost from *A. adenophora* negatively affected seed germination and the lengths of shoots and roots in crops and weeds.

*Fallopia japonica* (Houtt.) Ronse Decr., one of the 100 worst invasive species in the world [10], is native to Japan, North and South Korea, Taiwan and China but was introduced to Europe and North America for ornamental purposes during the early 19th century [11]. The success of the species has been partially attributed to its high tolerance to a wide range of conditions, including soil type and pH, salinity and drought [12,13,14]. The secondary metabolites from *F. japonica* rhizomes directly affect soil fauna through repellent and toxic properties and indirectly by influencing the soil biota [15].

The invasive herb *Solidago gigantea* Ait. is native to North America but was introduced to Europe and Asia as an ornamental plant. It negatively affects native communities outside its native range by decreasing species richness and diversity, apparently due to its intense competitive effects, rapid growth, or polyploidisation [16,17,18]. *S. gigantea* has broad tolerance to the levels of soil moisture, light, nutrient contents, temperature and soil pH [19]. *S. gigantea* produces a large amount of biomass, but this biomass contains few nutrients, decreases pH soil and the abundance of soil bacteria, and increases the C:N ratio and soil fungal biomass [20,21,22].

Soil ecologists have increasingly turned their attention to the effects of invasive plant species on soil community structure. Soil nematode communities, due to their abundance, diversity, and sensitivity to environmental changes, are considered excellent indicators of soil health [23,24]. Recent studies conducted under natural conditions have shown that the invasion of *F. japonica* reduced the total abundance and number of nematode species, particularly affecting, with negative impacts, omnivores, plant parasites and root–fungal feeders, but the diversity index remained unaffected [25,26]. Conversely, *S. gigantea* invasion reduces nematode diversity while increasing their abundance, particularly that of herbivores [27]. Quist et al. (2014) [28] and Harkes et al. (2021) [29] reported higher abundances of fungivores in the Aphelenchoididae and Aphelenchidae families at sites invaded by *S. gigantea*. The authors attributed these changes to a higher abundance of soil micromycetes, which are food for fungivore nematodes. On the other hand, the abundances of bacterivores, omnivores and predators were only slightly affected by the invasion of *S. gigantea* [27,28]. The influence of invasive *F. japonica* and *S. gigantea* on the structure of soil nematofauna has nevertheless been insufficiently investigated and whether the observed effects are due to invasive plants or differences in environmental conditions between invaded and uninvaded localities is not clear. The use of pot experiments is more advantageous than natural conditions in providing a better trial control, because the structure of soil nematode communities in natural environments is influenced not only by abiotic conditions (e.g., moisture content, temperature, pH and salinity) but also by a wide range of biotic conditions (e.g., vegetation and faunal composition, availability of food, presence of predators, parasites and diseases) [30,31].

The aim of this study was to investigate the impact of organic matter from two invaders, *F. japonica* and *S. gigantea*, on the selected soil parameters and structure of soil nematode communities in controlled conditions. We hypothesised the following: (1) The introduction of organic matter from *F. japonica* into the soil will decrease the total number of identified nematode genera and the total nematode abundance. The diversity of nematode communities will remain unaffected. (2) The introduction of organic matter from *S. gigantea* into the soil will cause an increase in nematode abundance, particularly herbivores. Additionally, there will be a decrease in the overall diversity of nematode communities. (3) The effects of adding organic matter from the two different invasive species on nematode communities will be different and species-specific. (4) We expected that the impact of decomposed organic matter from the invasive species on nematode communities would differ from the influence of live invaders. This difference would indicate specific effects of decomposed organic matter on the structure of nematode communities. (5) Differences in the structure of nematode communities caused by organic matter from two invaders in controlled conditions will be primarily attributed to the alteration in soil conditions caused by its addition, rather than variable in the environmental conditions between invaded and uninvaded localities in natural environments.

## 2. Results

### 2.1. Soil Physical and Chemical Properties

The soil physical properties, soil moisture content and pH differed significantly (Kruskal–Wallis test, *p* < 0.001) amongst the treatments (Table 1). The mean pH was highest (7.6) in S and lowest (6.3) in OMS and OMF. The mean pH in S/OMF and S/OMS was 7.1 and 7.0, respectively. These values were higher than those in OMF and OMS but lower than that in S. Multiple post hoc comparisons confirmed that the soil moisture content was significantly higher only in OMS and lower in S/OMF than in the other treatments (*p* < 0.001).

The measured soil chemical properties, the contents of organic carbon and total nitrogen were higher in mixed S/OMF and S/OMS than OMF, OMS and S (*p* < 0.01) (Table 1).

### 2.2. Total Number of Genera and Composition, Abundance, Diversity Index and Biomass of the Nematode Communities

The total numbers of identified nematode genera were 41 in S, 40 in S/OMF, 35 in S/OMS, 29 in OMF and 25 in OMS. The dominant genera (>5%) in S were *Rhabditis*, *Helicotylenchus*, *Filenchus*, *Tylencholaimus*, *Paratylenchus*, *Rotylenchus* and *Aphelenchus*. The dominant genera in S/OMF were *Rhabditis*, *Acrobeloides*, *Filenchus*, *Tylencholaimus*, *Pelodera* and *Eucephalobus*. The dominant genera in S/OMS were *Rhabditis*, *Filenchus*, *Acrobeloides*, *Tylencholaimus*, *Aphelenchoides* and *Helicotylenchus.* The dominant genera in OMF were *Rhabditis*, *Eudorylaimus*, *Acrobeloides*, *Tripyla*, *Pelodera* and *Eucephalobus.* The dominant genera in OMS were *Rhabditis*, *Tripyla*, *Acrobeloides* and *Aphelenchoides* (Table 2).

The total abundance of nematodes did not differ significantly amongst the treatments (Table 3), but decreased in all treatments during the study period (May to September), except in OMF, where the abundance was lowest in May and varied in the subsequent months (*p* < 0.05) (Table 4). The diversity index calculated for the genera (H’gen) was significantly higher in S and S/OMF than in OMF and OMS. H’gen, however, decreased during the study period both in S and S/OMF. H’gen was significantly lower in OMS than in S during all of the months studied (*p* < 0.05). It was lower in OMF than in S in June, July and August (*p* < 0.05). The total nematode biomass was higher in OMS than in S/OMF (*p* < 0.05), but decreased during the study in all treatments. The total biomass was significantly higher in May than at the end of the experiment in most of the treatments, except for OMF. In OMF, the total biomass did not vary significantly during the experiment period.

### 2.3. Ecological and Functional Indices of Nematode Communities

The Maturity Index was significantly lower in OMS than in S, S/OMF and S/OMS (*p* < 0.001). The Plant Parasitic Index did not differ amongst the treatments, but increased during the season in S and S/OMF (*p* < 0.05). In May and June, the Enrichment Index was significantly higher in OMF and OMS than in S (*p* < 0.001 and *p* < 0.01). In the same months, the Structural Index was significantly lower in OMF and OMS than in S. In September, it was higher in OMF than in S. The Channel Index was significantly lower in OMF and OMS than in S, S/OMF and S/OMS. The Basal Index was significantly lower in OMF than in the other treatments, but decreased during August and September compared to that in the S treatment. The Basal Index also decreased in OMS during May and June compared to that in S (Table 3 and Table 4).

### 2.4. Nematode Abundance in the Trophic and cp Groups

Bacterivores were the most abundant trophic group in all treatments. Their abundance was significantly higher in S/OMS and OMS at the beginning of the experiment in May (*p* < 0.001). Bacterivore abundance varied in S and OMF throughout the study and decreased significantly in S/OMS, S/OMF and OMS from May to September. Herbivores were the second most abundant trophic group in S, but they were very rare (<1%) in OMF and OMS. Herbivore abundance was similar in S/OMS and significantly lower in S/OMF compared to S (both *p* < 0.001). Fungivore abundance was low in OMF and OMS (*p* < 0.001) and it did not differ significantly amongst S, S/OMF, S/OMS. Omnivore abundance was significantly lower in S/OMS and OMS than in S, S/OMF and OMF (*p* < 0.001). The abundance of omnivores decreased in S and S/OMS and increased in OMF from May to September. Predator abundance did not differ significantly amongst the treatments, but increased in OMF and OMS during the study period, peaking in September (*p* < 0.05).

The total abundance of stress-tolerant c-p1 nematodes did not differ amongst the treatments, but gradually decreased from May to September in S/OMF, S/OMS and OMS (*p* < 0.05). The abundance of c-p2 nematodes was significantly higher in S/OMF, S/OMS and OMF in May than in September (*p* < 0.05). The abundance of c-p3 nematodes in OMF and OMS increased during the season, especially in August and September. The abundance of stress-sensitive c-p4 nematodes was lower in OMS (*p* < 0.001) and the abundance of c-p5 nematodes was lower in OMF and OMS than in S, S/OMF and S/OMS (*p* < 0.001) (Table 3 and Table 4).

## 3. Discussion

Invasive plants often produce significantly higher aboveground biomass at the end of the growing season compared to the native plant communities they displace [5]. This biomass, which often remains unnoticed at invaded sites, directly affects the soil ecosystem and serves as a source of food and habitat for a wide range of soil biota, including bacteria, fungi and invertebrates. In this study, we focus on the effect of the organic matter of two invaders, *F. japonica* and *S. gigantea*, on soil properties and nematode communities in controlled conditions.

### 3.1. OMF and S/OMF

OMF was characterised by higher moisture and organic carbon and low pH and nematode diversity index. Herbivores and c-p5 were very rare and fungivores were lower than in S. The Channel and Basal indices were lower than in the other treatments.

*F. japonica* has been reported to alter the soil’s pH [32,33], nutrient availability [34] and organic matter content [35] in natural conditions. These soil changes can subsequently affect the growth and activity of soil microorganisms such as bacteria and fungi, which play important roles in nutrient cycling and in the formation of soil structure. Moreover, they are also sources of food for bacterivores and fungivores. The root system of living *F. japonica* consists of strong rhizomes with only a few fine roots that reduce the availability of food and habitat for soil invertebrates, including nematodes [36]. Plots invaded by *F. japonica* in natural conditions had lower total abundances and fewer identified nematode species, with negative impacts mostly on herbivores, compared to uninvaded grassland, forests, or wetland [25,26]. We expected that adding OMF to the soil would strongly influence the abundance of basal trophic groups such as bacterivores and fungivores and that higher trophic levels, such as omnivores and predators, would be less influenced. The hypothesis that adding OMF to the soil would decrease the total abundance and number of identified nematode genera was not confirmed in our pot experiment. The decomposed organic matter of *F. japonica* had a specific effect that differed from the influence of the live invader. The organic matter of *F. japonica* has low quality, is nutritionally poor, and is rich in lignin [15]. The decomposition of *F. japonica* litter is therefore three- to four-fold slower than the decomposition of the litter of the original plants, which slows the cycling of organic substances in the soil [37]. Nevertheless, our results showed that in S/OMF, the total abundances of bacterivores and fungivores were similar to those in S, but decreased during the study period. The high content of antimicrobial substances such as tannins in the organic matter from *F. japonica* can be the cause of the decrease in the total biomass of soil microorganisms [37], which is reflected by the decrease in the abundances of bacterivores and fungivores in S/OMF in our study. In S/OMF, the herbivores were the most strongly influenced trophic group, whose abundance decreased nearly ten-fold (from 50 to 6 individuals per 100 g of soil) during the five months of the study. Because in all treatments we sowed a mixture of fast-growing grass to simulate the natural grassland conditions with strong root systems on which herbivores can feed, such a large reduction in the abundance of herbivores after the addition of OMF was probably caused by toxic secondary metabolites [15,38,39], with a direct negative effect on herbivores. The abundance of omnivores by the end of the experiment increased in OMF, but not in S/OMF. Čerevková et al. (2019) [25] observed a reduction in the abundance of omnivores in plots invaded by *F. japonica* compared to uninvaded forest, grassland, or wetland plots without changes in the abundance of predatory nematodes. In contrast, adding OMF to the soil increased the abundance of predators, especially the genus *Tripyla*, at the end of our experiment. This genus is common in diverse terrestrial habitats in moist soil with moss and their abundance is usually correlated with the presence of tree species, e.g., *Fagus sylvatica* [40]. Why its abundance rapidly increased via the application of OMF remains unknown.

In natural conditions, the Maturity, Channel and Structural indices were lower in localities invaded by *F. japonica* compared to uninvaded plots [25,26], but we did not find a similar pattern in the pot experiment.

### 3.2. OMS and S/OMS

OMS was characterised by the highest moisture and organic carbon values, and a low pH and diversity index. Herbivores, omnivores, c-p4 and c-p5 were very rare throughout the study period. The Maturity and Structure indices were lower than in the other treatments.

In S/OMS, we observed similar nematode abundance and diversity index compared to S. This finding did not correspond with previous results, such as those of Zhang et al. (2011) [41], who mentioned that secondary metabolites produced by *Solidago* can inhibit the growth and reproduction of soil organisms, including nematodes. Carson and Peterson (1990) [42] reported that the removal of *Solidago* litter from the invaded plots significantly increased the species richness of the plant communities. This could be attributed to the chemical and physical properties of the litter and was subsequently indicated by the diversity of the soil biota. On the other hand, Čerevková et al. (2020) [27] confirmed a higher nematode abundance at *Solidago*-invaded than uninvaded forest or grassland sites, and herbivorous nematodes were the most affected trophic group. The high nematode abundance in natural conditions was mostly due to the high abundance of the herbivores *Boleodorus*, *Geocenamus*, *Helicotylenchus*, *Paratylenchus* and *Rotylenchus*. This suggests that mostly monospecific stands of *S. gigantea* with rich root systems serve as food sources for herbivores and increase their abundance. In S/OMS, the herbivore abundance was similar compared to that in S. On the other hand, bacterivores and c-p1 enrichment opportunists were negatively affected; their abundances decreased dramatically during the study period in S/OMS and were lowest in OMS. Stress-tolerant c-p1 bacterivores are only active in transient conditions of high microbial activity [43] and their populations usually decrease due to dwindling supplies of microbial food [44]. Plots invaded by *S. gigantea* in natural conditions reduced the microbial activity [21,22] or altered the compositions of the soil microbial communities [45,46,47], analogously to the reduced abundance of bacterivores. Čerevková et al. (2020) [27] could not confirm a reduction in bacterivore abundance in natural conditions. Several studies [27,28,29] have reported increases in the abundance of fungivores in localities invaded by *S. gigantea* in natural conditions. The root system of *S. gigantea* promotes the growth of beneficial soil microorganisms, such as mycorrhizal fungi, which can increase abundance and diversity, especially for fungivores [48]. This positive effect on the abundance of fungivores in natural conditions was not confirmed in our experiment with organic matter from *S. gigantea*. Fungivore abundance increased only slightly in S/OMS, and in the OMS treatment, it was lower than in S. Nematode community structures in agricultural successional studies shift towards a higher representation of fungivores and the gradual reappearance of K-selected taxa (c-p3 to c-p5), including omnivores and predators [44,49,50], as nutrients become depleted. The abundance of opportunist c-p2 nematodes was higher in S/OMS than in the other treatments. This group was represented in S/OMS mainly by small tylenchids feeding on epidermal cells (*Filenchus*), aphelenchoid fungivores (*Aphelenchoides*) and cephalobid bacterivores (*Acrobeloides* and *Cephalobus*). Nematode c-p2 taxa are tolerant to disturbances and occur in food-rich and -poor conditions [43]. The results from the pot experiment confirmed a significantly lower abundance of omnivores in S/OMS and OMS than in S, S/OMF and OMF, respectively. The abundances of four omnivore genera were low in OMS (*Dorylaimus*, *Enchodelus*, *Eudorylaimu*s and *Mesodorylaimus*) and high in S/OMS only for the genus *Eudorylaimus*. The total abundance of predatory nematodes in our experiment did not differ amongst the treatments, especially because of the very high variability during the study. The abundance of predators, however, increased in both the S/OMS and OMS treatments during the study period, especially in the last month. The abundances of seven predatory genera were similar in S/OMS and the abundance of *Tripyla* was high in OMS.

An invasion by *S. gigantea* did not significantly affect the ecological and functional indices in natural conditions [27] and the use of organic matter from this invader in our pot experiment confirmed small or no differences in the indices.

## 4. Material and Methods

### 4.1. Experimental Plots, Litter and Soil Sampling

Biomasses were collected from abandoned locations around the Ružín water reservoir in Eastern Slovakia. This region has a moderately warm climate, with an average annual temperature of 6–8 °C and an average annual rainfall of 700–800 mm. The soil is classified as a Cambisol [51,52]. Standing aboveground plant biomass was collected from sites heavily invaded by *F. japonica* next to a stream in the village of Opátka (48°48.6784′ N, 21°03.4846′ E) and by *S. gigantea* in a field along a road in the village of Košická Belá (48°48.0269′ N, 21°06.4564′ E) in September 2020, two years before the experiment. We harvested standing aboveground plant biomass from five 1 × 1 m areas for each plant. Aboveground biomass was used to produce organic matter for our greenhouse experiment. Plants were cut using a hand sickle and transferred to the laboratory in separate plastic bags. Leaves, flowers and stems of the two species were subsequently chopped into small pieces and placed into two plastic garden composters (one for each species), in which a commercial accelerator with active plant ingredients (Orgamin, Forestina s. r. o.) was added to speed up the decomposition. We practised cold composting by adding pure, cut plant material to the pile, sprinkling it with water, and waiting two years to decompose. After two years of maturing in the composters, the decomposed organic matter was used for the pot experiment. Soil for the pot experiment was collected from the upper soil layer (15 cm) from sites of permanent grasslands with indigenous plant species. Soil samples were carefully mixed to create an average sample with homogenised nematode communities.

**Figure 1:**
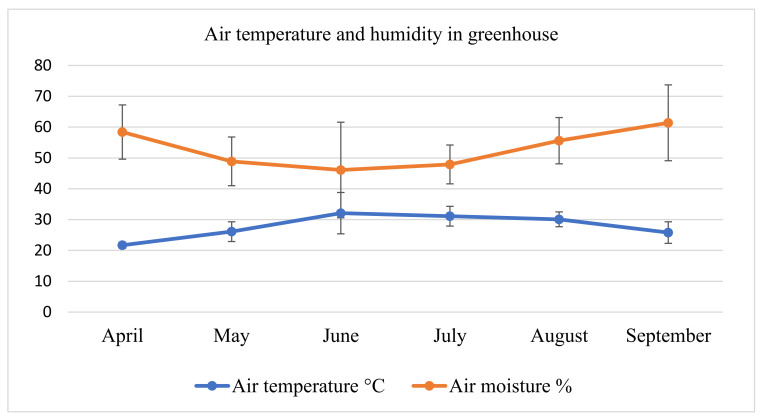
Mean temperature (°C) and air humidity (%) measured in greenhouse every working day at the same time ± standard deviation.

The pot experiment began on 24 April 2022 in a greenhouse where air temperature and humidity were regularly checked at the same time every working day (Figure 1). The pot experiment consisted of five treatments arranged in five replicates. (1) S, consisting of 25 pots of mixed soil. (2) S/OMF, consisting of 25 pots with 50% soil plus 50% organic matter from *F. japonica*. (3) S/OMS, consisting of 25 pots with 50% soil plus 50% organic matter from *S. gigantea*. (4) OMF, consisting of 25 pots with 100% organic matter from *F. japonica*. (5) OMS, consisting of 25 pots with 100% organic matter from *S. gigantea*. Each pot with a volume of 2.5 L was fully filled with the treatment. All the contents in the pots were thoroughly mixed. There were five replications for each treatment representing five months of the growing season (May to September). A total of 125 pots were prepared (five treatments × five replicates × five months).

**Figure 2:**
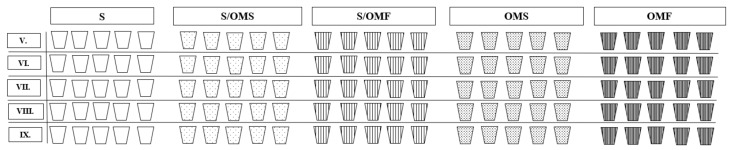
Graphic scheme of the pot experiment using five treatments: (i) ‘soil’—S, (ii) 50% soil plus 50% decayed organic matter of *Fallopia japonica* S/OMF, (iii) 50% soil plus 50% of *Solidago gigantea* S/OMS, (iv) 100% decayed organic matter of *F. japonica* OMF and (v) 100% decayed organic matter of *S. gigantea* OMF. The experiment was carried out for five months from May to September (V.–IX.).

To simulate natural grassland conditions, on the surface of each pot, we sowed 2 g of a commercial mixture of fast-growing grass seed suitable for meadows and pastures without meadow flowers (Lúčna, Optima, Aquaseed s.r.o., Košice, Slovakia). The pots were watered three times a week and the vegetation trimmed using grass shears as needed. The first sampling was carried out seven days after the established pot experiment in order to explore the initial nematode communities’ compositions. The samples were collected on 2 May, 6 June, 11 July, 15 August and 12 September 2022 (Figure 2). The samples were put into plastic bags and labelled according to the treatment, month of sampling and pot number, and were then transferred to the laboratory for analysis.

### 4.2. Soil Properties

The soil moisture content was measured from 100 g of all samples of each treatment every month. The content of the fresh samples was estimated gravimetrically via oven-drying at 105 °C for 24 h. The chemical properties were analysed in air-dried samples. The pH was measured in May and June from 10 g of three samples from each treatment. The pH was measured potentiometrically in a water suspension using an HI99121 digital pH meter at a 1:2 soil:KCl (HI7051L) ratio. The samples for chemical analysis were air-dried and completely crushed in a porcelain crucible. The contents of organic carbon and total nitrogen were determined using Turin’s method [53] and were measured by the Soil Science and Conservation Research Institute in Slovakia.

### 4.3. Nematode Extraction, Identification and Evaluation

One hundred grams of samples from each pot were processed to extract nematodes using a combination of Cobb sieving and a modified Baermann’s technique [54]. Nematodes in aqueous suspensions were killed in a warm water bath (70 °C) and counted using a Leica S8APO stereomicroscope (Germany) at magnifications up to 80×. Nematodes were microscopically identified at the genus level using an Eclipse 90i light microscope (Nikon, Japan) at magnifications of 100, 200, 400 and 600×. A minimum of 100 individuals were identified in each sample. Nematode abundance was expressed as the number of individuals per 100 g of dry soil. The Shannon–Weaver index for genera (H’gen) was used for calculating generic diversity, H’gen = −∑(Pi × lnPi), where Pi is the proportion of the genus divided by the total nematode abundance in the sample [55]. The Maturity Index measures environmental disturbance and is calculated using coloniser–persister (c-p) classes [23]. These classes represent the life-history characteristics of the taxa associated with r- and K-selection. Species with c-p values of 1 or 2 are r-selected, i.e., colonisers, and species with a c-p value of 5 are K-selected, i.e., persisters. The Maturity Index (MI) calculated for free-living taxa and Plant Parasitic Index (PPI) calculated for herbivores were calculated as *XI* = ∑ [*vi* × *fi*)]/n, where *XI* is the index of interest (MI or PPI), *vi* is the coloniser–persister (c-p) value of taxon *i*, *fi* is the frequency of taxon *i* in a sample and *n* is the total number of individuals in the same sample [23]. We calculated the indices of the soil food web, namely the Channel, Basal, Enrichment and Structural indices, according to the work of Ferris et al. (2001) [56]. All indices and nematode total biomass were calculated using the online programme Nematode Indicator Joint Analysis (beta) (https://shiny.wur.nl/ninja/ Accessed 22 April 2023) [57]. Nematode genera were assigned to trophic groups (herbivores, fungivores, bacterivores, predators, and omnivores) according to the work of Yeates et al. (1993) and Wasilewska (1997) [58,59], and were assigned to c-p groups following the research of Ettema and Bongers (1993) [60].

### 4.4. Statistical Analysis

All statistical analyses were performed using the PAST 4.03 statistical programme [61]. Data were log + 1 transformed prior analysis. The nonparametric Kruskal–Wallis test was used to identify significant differences in the variables evaluated amongst the treatments (S, S/OMF, S/OMS, OMF and OMS). For each treatment, the nonparametric Friedman test (for dependent measurements) was used to identify significant differences in the variables evaluated amongst the months. The findings of the Kruskal–Wallis and Friedman tests were considered to be significant at *p* < 0.05 (*), <0.01 (**) and <0.001 (***).

## 5. Conclusions

Our results suggest that the experimental addition of decayed organic matter from two invasive plants to the soil affected the soil nematode communities, and the nature of this impact depended on the invasive plant species. The low quality of organic matter from *F. japonica*, with high lignin and tannin contents, or the organic matter from *S. gigantea*, with few nutrients that decrease the soil pH and increase the C:N ratio, may have led to a decrease in productivity and slow nutrient cycling, demonstrated by the decrease in the abundance of bacterivores in both S/OMS and S/OMF. Invasive plants, therefore, not only reduce the soil’s diversity, but their organic matter left on the land can also reduce the soil’s quality, which is detrimental to nematodes and decreases the diversity of the nematode community. A reduction in the abundance of herbivores, especially after the addition of organic matter from *F. japonica* to the soil, could potentially be used as an alternative method to protect plants against plant parasitic nematodes, although further studies in this area are needed.

## Figures and Tables

**Table 1:** Mean value of measured parameters: pH, moisture, organic carbon, total nitrogen and standard deviation (A ± SD) in five tested treatments: (i) ‘soil’—S, (ii) 50% soil plus 50% decayed organic matter of *Fallopia japonica* S/OMF, (iii) 50% soil plus 50% of *Solidago gigantea* S/OMS, (iv) 100% decayed organic matter of *F. japonica* OMF and (v) 100% decayed organic matter of *S. gigantea* OMS.

Measured Parameters	S	S/OMF	S/OMS	OMF	OMS	*p*
pH	7.6 ± 0.1 a	7.1 ± 0.1 b	7.0 ± 0.0 b	6.3 ± 0.5 c	6.3 ± 0.5 c	***
Moisture	29.6 ± 7.3 a	24.2 ± 9.2 b	28.4 ± 12.5 ab	31.4 ± 7.4 ac	36.5 ± 9.5 c	***
Organic carbon	7.2 ± 0.65 a	15.0 ± 0.97 b	14.8 ± 0.50 b	9.15 ± 0.12 c	9.35 ± 0.46 c	**
Total nitrogen	1.11 ± 0.06 a	1.79 ± 0.08 b	1.81 ± 0.14 b	1.19 ± 0.06 a	1.32 ± 0.09 ab	**

Kruskal–Wallis test results (** *p* < 0.01; *** *p* < 0.001) expressing significant differences among the five tested treatments: S, S/OMF, S/OMS, OMF and OMS. Means with the same superscript small letter (a, b, c) are not significantly different based on the least significant difference tests (*p* < 0.01) (n = 25).

**Table 2:** List of identified nematode genera, mean abundance and standard deviation (A ± SD) and dominance (D%) in five tested treatments: (i) ‘soil’—S, (ii) 50% soil plus 50% decayed organic matter of *Fallopia japonica* S/OMF, (iii) 50% soil plus 50% of *Solidago gigantea* S/OMS, (iv) 100% decayed organic matter of *F. japonica* OMF and (v) 100% decayed organic matter of *S. gigantea* OMS.

		S		S/OMF		S/OMS		OMF		OMS	
Nematode Genera	c-p	A ± SD	D%	A ± SD	D%	A ± SD	D%	A ± SD	D%	A ± SD	D%
Bacterivores											
*Acrobeles*	2	0.1 ± 0.4	0.07	0.1 ± 0.26	0.04	0.1 ± 0.39	0.06	0.2 ± 0.92	0.14	0.3 ± 1.12	0.19
*Acrobeloides*	2	6.8 ± 4.95	3.99	14.2 ± 12.09	9.69	19.3 ± 19.93	10.74	16 ± 16.28	12.31	12 ± 14.03	8.11
*Alaimus*	4	2.5 ± 3.68	1.5	0.3 ± 0.83	0.22	0.9 ± 2.05	0.5	0.2 ± 0.49	0.14	-	-
*Aulolaimus*	3	-	-	0 ± 0.24	0.03	-	-	-	-	-	-
*Cephalobus*	2	8.5 ± 11.89	4.98	4.3 ± 4.83	2.92	3.6 ± 6.26	2.01	0.4 ± 0.82	0.32	1.5 ± 2.75	1.04
*Cervidellus*	2	0.2 ± 0.6	0.12	1 ± 2.11	0.66	0.4 ± 0.69	0.2	1.9 ± 3.44	1.47	1 ± 1.77	0.7
*Diploscapter*	1	0.4 ± 0.96	0.25	0.3 ± 0.83	0.17	1.3 ± 2.31	0.73	1 ± 3.46	0.78	0.8 ± 2.42	0.55
*Eucephalobus*	3	7.5 ± 6.84	4.44	10.4 ± 10.25	7.12	8.2 ± 6.18	4.57	6.9 ± 8.1	5.32	2.1 ± 2.25	1.44
*Chiloplacus*	2	0 ± 0.24	0.03	0.1 ± 0.32	0.04	1.1 ± 2.75	0.6	-	-	-	-
*Monhystera*	2	-	-	0.1 ± 0.32	0.06	-	-	0.1 ± 0.26	0.04	-	-
*Panagrolaimus*	1	0.1 ± 0.34	0.04	0.1 ± 0.34	0.05	-	-	-	-	-	-
*Pelodera*	1	3.4 ± 4.6	2.01	10.9 ± 17.07	7.44	4.6 ± 6	2.56	7.1 ± 11.1	5.5	4.6 ± 7.07	3.1
*Plectus*	2	2.7 ± 2.87	1.58	2.6 ± 3.12	1.77	1.4 ± 2.14	0.8	1 ± 1.16	0.77	0.7 ± 1.39	0.45
*Prismatolaimus*	3	0.4 ± 0.77	0.21	0.5 ± 2.35	0.36	0.4 ± 1.02	0.23	0.1 ± 0.26	0.04	0 ± 0.24	0.03
*Protorhabditis*	1	-	-	0.2 ± 0.5	0.12	1.2 ± 3.23	0.68	-	-	-	-
*Rhabditis*	1	30.6 ± 16.5	18	28 ± 21.42	19.16	48.9 ± 54.5	27.14	54.4 ± 35.08	41.89	82.8 ± 89.41	55.87
*Wilsonema*	2	0.2 ± 0.58	0.15	0.2 ± 0.46	0.11	0.1 ± 0.55	0.08	0.1 ± 0.4	0.09	-	-
Fungivores											
*Aphelenchoides*	2	1.8 ± 2.08	1.08	6.5 ± 5.42	4.43	13.2 ± 10.49	7.32	3.7 ± 3.36	2.84	10.7 ± 8.73	7.2
*Aphelenchus*	2	9.4 ± 8.85	5.51	6.2 ± 4.5	4.22	5.4 ± 4.79	3	1.7 ± 4.78	1.28	0.9 ± 1.76	0.63
*Diphtherophora*	3	1.6 ± 1.61	0.93	0.7 ± 1.39	0.48	0.9 ± 2.68	0.52	0.5 ± 1.33	0.36	-	-
*Filenchus*	2	11.8 ± 19.77	6.96	11.3 ± 17.91	7.7	19.5 ± 25.21	10.85	1.6 ± 3.01	1.2	6.6 ± 12.41	4.44
*Tylencholaimus*	4	11.4 ± 6.92	6.7	11.3 ± 13.67	7.7	13.9 ± 18.74	7.69	0.1 ± 0.28	0.04	0.1 ± 0.44	0.09
Herbivores											
*Aglenchus*	2	0.3 ± 1.72	0.2	-	-	-	-	-	-	-	-
*Axonchium*	5	-	-	-	-	0 ± 0.24	0.03	-	-	-	-
*Boleodorus*	2	0.7 ± 1.73	0.41	0.3 ± 0.74	0.17	-	-	-	-	-	-
*Criconema*	3	1.6 ± 2.55	0.93	1 ± 2.2	0.69	0.4 ± 1.29	0.23	-	-	-	-
*Helicotylenchus*	3	23.7 ± 10.65	13.94	7.2 ± 7.19	4.89	9.2 ± 10.35	5.12	0.1 ± 0.39	0.09	0.2 ± 0.58	0.14
*Heterodera*	3	2.6 ± 6.56	1.54	1.1 ± 3.82	0.77	1 ± 1.86	0.55	-	-	-	-
*Paratylenchus*	2	10.9 ± 10.03	6.39	5.3 ± 8.23	3.62	4.8 ± 5.41	2.66	0.1 ± 0.37	0.08	0.1 ± 0.48	0.09
*Pratylenchoides*	3	-	-	-	-	0.1 ± 0.34	0.04	-	-	-	-
*Pratylenchus*	3	0.4 ± 1.18	0.26	0.7 ± 1.49	0.48	0.3 ± 1.11	0.19	0.1 ± 0.5	0.11	0.3 ± 1.12	0.19
*Rotylenchus*	3	10 ± 7.85	5.91	1.7 ± 1.67	1.15	3.6 ± 4.01	1.99	-	-	0.1 ± 0.34	0.05
*Trichodorus*	4	0.4 ± 1.63	0.26	-	-	0.2 ± 0.72	0.11	0.1 ± 0.28	0.04	-	-
*Tylenchorhynchus*	3	8.4 ± 11.76	4.93	2.3 ± 3.03	1.57	7.8 ± 8.59	4.35	-	-	-	-
*Tylenchus*	2	0.1 ± 0.34	0.04	0.2 ± 0.59	0.11	-	-	-	-	-	-
Omnivores											
*Discolaimus*	4	-	-	-	-	-	-	0.1 ± 0.28	0.04	-	-
*Dorylaimus*	4	0.1 ± 0.34	0.04	-	-	-	-	-	-	-	-
*Enchodelus*	4	0.7 ± 1.56	0.44	0.3 ± 0.77	0.2	-	-	0.1 ± 0.28	0.04	0.1 ± 0.3	0.04
*Eudorylaimus*	4	6.2 ± 7.18	3.63	6.9 ± 9.11	4.7	1.9 ± 3.09	1.05	19.6 ± 28.47	15.11	0.9 ± 1.75	0.61
*Mesodorylaimus*	4	0.2 ± 0.73	0.14	0.1 ± 0.26	0.04	-	-	-	-	0.1 ± 0.36	0.05
Predators											
*Anatonchus*	4	0.1 ± 0.32	0.04	0.1 ± 0.44	0.09	-	-	-	-	0.1 ± 0.42	0.08
*Clarkus*	4	0.1 ± 0.74	0.09	0.1 ± 0.26	0.04	0.1 ± 0.53	0.08	0.2 ± 0.69	0.18	0.3 ± 0.72	0.22
*Iotonchus*	4	0.1 ± 0.32	0.04	-	-	-	-	-	-	-	-
*Mylonchulus*	4	0.9 ± 1.35	0.54	0.5 ± 1.43	0.32	0.1 ± 0.26	0.03	-	-	-	-
*Oxydirus*	5	1.8 ± 1.43	1.09	2.4 ± 3.38	1.62	1.4 ± 2.44	0.75	0.1 ± 0.3	0.05	0.1 ± 0.72	0.1
*Thonus*	4	0.8 ± 2.06	0.49	1.5 ± 4.37	1.03	0.6 ± 1.44	0.33	1.2 ± 2.94	0.89	-	-
*Tripyla*	3	0.2 ± 0.51	0.11	5.9 ± 12.54	4.01	4 ± 8.01	2.21	11.5 ± 15.07	8.83	21.6 ± 32.68	14.59
Number of genera		**41**		**40**		**35**		**29**		**25**	

**Table 3:** Means (A) and standard deviation (SD) of nematode abundance, number of identified genera, diversity index for genera, total biomass, abundance nematodes in trophic groups and coloniser–persister (cp and pp) groups, ecological and functional indices in five different treatments: (i) ‘soil’—S, (ii) 50% soil plus 50% decayed organic matter of *Fallopia japonica* S/OMF, (iii) 50% soil plus 50% of *Solidago gigantea* S/OMS, (iv) 100% decayed organic matter of *F. japonica* OMF, and (v) 100% decayed organic matter of *S. gigantea* OMS.

Index Name	S	S/OMF	S/OMS	OMF	OMS	
	A ± SD	A ± SD	A ± SD	A ± SD	A ± SD	*p*
Abundance, ind.	169 ± 53.8 a	146 ± 77.2 a	180 ± 104 a	129 ± 34.5 a	148 ± 84.8 a	-
Number of genera	17.9 ± 3.93 a	16.1 ± 5.70 a	14.9 ± 4.89 a	8.48 ± 1.72 b	7.68 ± 1.57 b	***
Diversity index for genera	1.0 ± 0.1 a	1.0 ± 0.2 a	0.9 ± 0.2 ab	0.6 ± 0.1 b	0.5 ± 0.1 b	***
Total biomass, mg	0.5 ± 0.4 ab	0.4 ± 0.3 a	0.5 ± 0.5 ab	0.6 ± 0.3 ab	0.8 ± 0.7 b	*
Maturity Index	2.2 ± 0.3 a	2.2 ± 0.5 a	2.1 ± 0.5 a	1.9 ± 0.7 ab	1.7 ± 0.8 b	***
Plant Parasitic Index	2.8 ± 0.2 a	2.8 ± 0.2 a	2.8 ± 0.2 a	2.9 ± 0.6 a	2.8 ± 0.2 a	-
Channel Index	16.2 ± 14.9 ab	20.1 ± 14.9 a	20.2 ± 14.4 a	4.2 ± 6.5 c	14.5 ± 22.7 b	***
Basal Index	15.9 ± 6.1 ab	15.7 ± 7.1 ab	18.9 ± 9.7 a	10.2 ± 9.6 c	13.8 ± 15.7 bc	***
Enrichment Index	75.3 ± 9.28 ab	71.5 ± 13.9 a	70.7 ± 16.0 a	85.9 ± 11.3 b	82.6 ± 17.5 ab	**
Structure Index	66.8 ± 12.2 a	65.5 ± 16.8 ab	51.2 ± 23.5 ab	50.3 ± 36.8 ab	42.2 ± 30.9 b	*
Herbivores ind.	59.2 ± 24.5 a	19.7 ± 20.3 b	27.4 ± 18.1 ab	0.4 ± 0.8 c	0.7 ± 1.7 c	***
Fungivores ind.	35.9 ± 19.6 a	35.9 ± 29.1 ab	52.9 ± 29.2 a	7.4 ± 8.8 c	18.3 ± 11.3 bc	***
Bacterivores ind.	63.5 ± 27.5 a	73.1 ± 45.8 a	91.7 ± 71.9 a	89.6 ± 45.6 a	105.0 ± 87.8 a	-
Predators ind.	4.0 ± 3.21 a	9.2 ± 11.7 a	5.5 ± 7.7 a	11.8 ± 14.6 a	22.2 ± 31.6 a	-
Omnivores ind.	7.3 ± 8.0 a	8.4 ± 9.6 a	2.5 ± 4.1 b	20.8 ± 28.2 a	1.0 ± 1.7 b	***
c-p1 ind.	34.5 ± 16.9 a	39.4 ± 33.7 a	56.1 ± 59.6 a	62.5 ± 41.1 a	88.2 ± 90.2 a	-
c-p2 ind.	49.0 ± 23.9 ab	56.6 ± 40.6 ab	72.3 ± 43.4 b	33.3 ± 21.2 a	35.6 ± 19.9 a	***
c-p3 ind.	2.1 ± 1.72 a	7.14 ± 12.2 a	5.34 ± 8.11 a	11.9 ± 15.0 a	21.6 ± 31.9 a	-
c-p4 ind.	23.2 ± 13.9 a	20.9 ± 15.3 a	17.4 ± 17.9 a	21.3 ± 28.1 a	1.60 ± 1.72 b	***
c-p5 ind.	1.9 ± 1.4 a	2.4 ± 3.3 a	1.4 ± 2.4 abc	0.1 ± 0.3 c	0.1 ± 0.7 bc	***

Kruskal–Wallis test results (* *p* < 0.05; ** *p* < 0.01; *** *p* < 0.001) expressing significant differences among the five tested treatments: S, S/OMF, S/OMS, OMF and OMS. Means with the same superscript small letter (a, b, c) are not significantly different based on the least significant difference tests (*p* < 0.01) (n = 25).

**Table 4:** Comparison of mean values of nematode abundance, number of identified genera, diversity index for genera, total biomass, ecological and functional indices, trophic and c-p groups in the five different treatments: (i) ‘soil’—S, (ii) 50% soil plus 50% decayed organic matter of *Fallopia japonica* S/OMF, (iii) 50% soil plus 50% of *Solidago gigantea* S/OMS, (iv) 100% decayed organic matter of *F. japonica* OMF, and (v) 100% decayed organic matter of *S. gigantea* OMS five months (from May to September) for each variant and among five variants during each month.

Index Name	S			S/OMF			S/OMS			OMF			OMS		
	Mean	Rows	Columns	Mean	Rows	Columns	Mean	Rows	Columns	Mean	Rows	Columns	Mean	Rows	Columns
Total number, ind.	169			146			180			130			149		
V.	220	ab ^(1)^	A ^(2)^	264	ab	A	341	a	A	101	b	A	259	ab	A
VI.	212	a	A	158	a	AB	162	a	AB	160	a	A	165	a	A
VII.	143	ab	AB	149	ab	AB	169	b	AB	114	ab	A	70.5	a	B
VIII.	154	a	AB	72.2	a	B	126	a	B	132	a	A	127	a	AB
IX.	120	a	B	87.6	a	B	103	a	B	142	a	A	120	a	AB
Number of genera	17.9			16.1			18.7			8.5			8.1		
V.	23.2	ab	A	25.6	a	A	22.0	b	A	8.2	c	A	8.6	c	A
VI.	20.0	a	AB	16.4	b	AB	15.0	b	AB	9.4	c	A	8.6	c	A
VII.	17.0	a	BC	15.8	ab	BC	14.8	b	B	8.8	c	A	8.6	c	A
VIII.	15.4	a	BC	10.8	b	C	10.2	bc	B	8.2	c	A	7.6	c	A
IX.	14.2	a	C	12.0	ac	BC	12.8	ac	B	7.8	bc	A	7.0	b	A
Diversity index	1.0			1.0			0.9			0.6			0.5		
V.	1.2	ab	A	1.2	b	A	1.0	bc	A	0.6	ac	A	0.4	c	A
VI.	1.1	a	A	0.9	ab	B	0.9	ab	A	0.6	b	A	0.4	b	A
VII.	1.0	a	AB	1.0	ab	AB	0.9	ac	A	0.7	bc	A	0.5	c	A
VIII.	0.9	a	BC	0.8	ab	B	0.9	ab	A	0.5	b	A	0.6	b	A
IX.	0.9	a	C	0.9	a	B	0.8	ab	A	0.6	ab	A	0.6	b	A
Total biomass, mg	0.5			0.4			0.5			0.6			0.8		
V.	0.9	ab	A	0.7	ab	AB	1.4	ab	A	0.5	b	A	1.8	a	A
VI.	0.4	a	AB	0.7	ac	B	0.5	ab	B	0.9	bc	A	1.1	c	AB
VII.	0.3	a	B	0.2	a	C	0.3	a	BC	0.4	a	A	0.2	a	C
VIII.	0.4	a	AB	0.3	a	AC	0.2	a	BC	0.6	a	A	0.6	a	BC
IX.	0.3	ab	B	0.3	ab	AC	0.2	a	C	0.6	b	A	0.6	ab	BC
Maturity Index	2.2			2.2			2.1			2.0			1.7		
V.	2.3	ab	A	2.1	ac	A	1.6	bc	A	1.3	bc	AB	1.2	b	A
VI.	2.3	a	A	1.6	ab	A	1.7	ab	AB	1.3	b	B	1.2	b	A
VII.	2.3	a	A	2.5	a	B	2.0	a	BC	1.9	a	A	1.8	a	B
VIII.	2.0	a	A	2.4	a	BC	2.2	a	C	2.4	b	A	1.9	a	BC
IX.	2.0	a	A	2.6	ab	C	2.8	b	D	2.8	b	A	2.3	ab	C
Plant Parasitic Index	2.8			2.8			2.9			2.8			2.8		
V.	2.6	a	A	2.6	a	A	2.7	a	A	3.0	a	A	2.7	a	A
VI.	2.7	ab	AC	2.7	ab	AB	2.8	b	A	2.0	ab	A	ND	a	A
VII.	2.9	a	B	2.8	ab	AB	2.9	ab	A	ND	a	A	3.0	ab	A
VIII.	2.9	a	BC	2.9	a	B	3.0	a	A	ND	a	A	ND	a	A
IX.	2.9	ab	BC	2.9	ab	B	3.0	b	A	3.3	ab	A	ND	a	A
Channel Index	16.2			20.1			20.2			4.2			14.5		
V.	20.8	ab	A	23.9	b	A	11.1	bc	A	1.2	c	A	2.5	ac	A
VI.	10.1	ab	A	2.9	ac	B	20.7	b	A	1.2	c	A	3.4	bc	A
VII.	14.2	a	A	32.4	a	A	21.4	a	A	10.5	a	A	51.6	a	B
VIII.	9.1	a	A	14.2	a	A	15.7	a	A	4.3	a	A	6.5	a	A
IX.	26.6	a	A	26.9	a	A	32.3	ab	A	3.9	b	A	8.8	ab	AB
Basal Index	15.9			15.7			18.9			10.2			13.8		
V.	16.3	ab	A	22.9	a	A	13.6	ab	A	9.90	ab	AB	4.13	b	A
VI.	17.8	a	A	9.61	ab	B	19.1	a	A	7.58	ab	AB	4.44	b	A
VII.	15.6	a	A	22.4	a	A	29.7	a	A	25.4	a	B	39.6	a	B
VIII.	11.5	ab	A	9.94	ab	B	18.9	a	A	4.19	b	A	11.8	ab	A
IX.	18.7	a	A	13.9	ab	AB	12.9	ab	A	4.00	b	A	8.89	ab	A
Enrichment Index	75.3			71.5			70.7			85.9			82.7		
V.	74.9	ac	A	67.5	a	AB	85.1	ab	A	89.8	bc	A	95.8	b	A
VI.	70.1	a	A	88.6	ab	A	79.1	ab	A	92.3	b	A	95.5	b	A
VII.	72.8	a	A	57.6	a	B	60.4	a	A	69.5	a	B	57.0	a	B
VIII.	84.2	a	A	80.3	a	AC	70.8	a	A	87.7	a	A	83.4	a	A
IX.	74.7	ab	A	63.4	ab	BC	58.2	a	A	90.3	b	A	81.5	ab	A
Structure Index	66.8			65.5			51.2			50.3			42.2		
V.	69.6	a	A	56.6	ab	A	37.8	ab	AB	9.11	b	A	19.9	b	A
VI.	69.1	a	A	53.5	ab	A	33.9	ab	AB	23.2	b	AB	32.5	ab	A
VII.	70.6	a	A	66.4	a	A	43.9	ab	A	38.9	ab	BC	9.69	b	B
VIII.	70.8	a	A	70.4	a	A	56.7	a	A	86.5	a	BC	64.1	a	AC
IX.	53.8	a	A	80.7	ab	A	83.8	ab	B	93.8	b	C	84.9	ab	C
Herbivores ind.	59.2			19.7			27.5			0.4			0.7		
V.	72.7	a	A	49.8	ab	A	40.3	ab	A	1.0	b	A	2.8	b	A
VI.	69.4	a	A	9.3	ab	BC	20.1	ab	A	0.3	b	A	0.0	b	A
VII.	69.6	a	A	26.4	ab	AB	37.6	ab	A	0.0	b	A	0.7	b	A
VIII.	46.4	a	A	6.7	ab	C	25.8	ab	A	0.0	b	A	0.0	b	A
IX.	37.9	a	A	6.2	ab	C	13.5	ab	A	0.8	b	A	0.0	b	A
Fungivores ind.	36.0			35.9			52.9			7.4			18.3		
V.	48.3	ab	A	77.5	a	A	81.6	a	A	3.73	b	A	19.8	ab	A
VI.	29.7	a	A	14.5	ab	B	62.8	a	A	4.66	b	A	18.6	ab	A
VII.	24.3	ab	A	55.5	b	A	37.8	ab	A	15.5	a	A	29.1	ab	A
VIII.	39.1	a	A	11.1	ab	B	35.7	a	A	6.51	b	A	13.3	ab	A
IX.	38.6	a	A	20.9	a	B	46.6	a	A	6.78	a	A	10.6	a	A
Bacterivores ind.	63.5			73.1			91.8			89.3			106.0		
V.	75.4	a	AB	115.0	ac	A	207.0	bc	A	94.9	ab	AB	232.0	c	A
VI.	93.3	ab	B	125	ab	A	76.9	b	B	152	b	B	143	b	AB
VII.	43.1	ab	A	60.9	ab	AB	88.9	b	AB	81.2	ab	AB	38.3	a	C
VIII.	64.0	a	AB	34.7	a	B	60.5	a	B	69.3	a	A	72.5	a	BC
IX.	41.6	a	A	29.2	a	B	24.4	a	C	48.8	a	A	43.2	a	C
Predators ind.	4.0			9.2			5.5			11.8			22.3		
V.	5.6	ab	AB	10.4	a	AC	3.7	ab	AB	0.3	b	A	1.4	ab	A
VI.	7.5	a	A	2.2	ab	B	1.0	b	A	2.5	ab	AB	3.7	ab	AB
VII.	3.4	a	B	3. 8	a	BC	2.3	a	AB	14.5	a	BC	2.2	a	A
VIII.	2.2	a	B	7.1	a	AB	4.0	a	AB	10.5	a	BC	38.5	a	BC
IX.	1.3	a	B	22.3	ab	A	16.7	ab	B	31.3	b	C	65.5	b	C
Omnivores ind.	7.3			8.4			2.5			20.8			1.0		
V.	18.1	a	A	10.9	ab	A	7.9	ac	A	0.3	c	A	1.1	bc	A
VI.	12.3	a	A	6.8	ab	A	0.7	ab	B	0.7	ab	A	0.0	b	A
VII.	3.1	a	B	2.9	a	A	2.2	a	B	3.1	a	A	0.3	a	A
VIII.	2.5	ab	B	12.6	ab	A	0.0	a	AB	46.1	ab	B	2.4	b	A
IX.	0.6	a	B	9.0	ab	A	1.5	ab	B	53.9	b	B	1.1	a	A
c-p1 ind.	34.5			39.4			56.1			62.5			88.2		
V.	39.9	a	A	50.1	ac	AB	158.0	bc	A	66.9	ab	AB	218.0	b	A
VI.	35.9	a	A	91.4	ab	B	58.3	ab	AB	120	b	B	135	b	AB
VII.	21.1	a	A	17.8	a	AC	29.1	a	B	35.4	a	A	15.9	a	C
VIII.	46.7	a	A	23.6	a	AC	29.8	a	B	54.7	a	AB	44.8	a	BD
IX.	28.8	a	A	14.2	a	C	5.35	a	C	35.4	a	A	27.1	a	CD
c-p2 ind.	49.0			56.6			72.3			33.3			35.6		
V.	66.9	ab	AB	124.0	b	A	121.0	ab	A	30.2	a	A	33.1	a	A
VI.	68.4	a	A	43.2	ab	BC	74.1	ab	A	36.9	ab	AB	26.3	b	A
VII.	31.8	a	B	67.9	ab	AB	85.2	b	A	61.3	ab	A	51.1	ab	A
VIII.	33.6	a	AB	20.9	a	C	49.4	a	AB	18.5	a	AB	40.7	a	A
IX.	41.3	a	AB	27.2	a	C	31.8	a	B	16.6	a	B	26.7	a	A
c-p3 ind.	2.1			7.14			5.34			11.9			21.6		
V.	2.0	ab	A	5.8	b	AB	5.4	ab	AB	0.0	a	A	0.4	ab	A
VI.	0.6	a	A	0.2	a	A	0.7	a	A	1.3	a	A	1.8	a	A
VII.	3.9	a	A	2.1	a	A	1.1	a	A	14.5	a	AB	2.2	a	A
VIII.	2.8	a	A	5.0	a	AB	2.6	a	A	12.9	a	AB	38.5	a	B
IX.	1.4	a	A	22.6	ab	B	16.9	ab	B	31.2	b	B	65.5	b	B
c-p4 ind.	23.2			20.9			17.4			21.3			1.60		
V.	36.6	a	A	27.9	ab	A	12.8	ac	A	0.9	c	A	1.4	bc	A
VI.	34.2	a	AB	11.9	ab	A	7.9	ab	A	1.9	b	A	2.1	b	A
VII.	15.2	ab	BC	33.8	b	A	14.9	ab	A	3.1	a	A	0.6	a	A
VIII.	20.4	ab	AC	13.9	ab	A	16.7	ab	A	46.4	a	B	2.7	b	A
IX.	9.3	ab	C	17.2	ab	A	34.8	ab	A	54.5	b	B	1.10	a	A
c-p5 ind.	1.9			2.4			1.4			0.1			0.1		
V.	1.6	a	A	6.4	a	A	3.7	a	A	0.3	a	A	0.7	a	A
VI.	3.3	a	A	1.6	ab	A	0.3	ab	A	0.0	b	A	0.0	b	A
VII.	1.8	a	A	1.4	a	A	0.9	a	A	0.0	a	A	0.0	a	A
VIII.	1.2	a	A	2.0	a	A	1.6	a	A	0.0	a	A	0.0	a	A
IX.	1.3	a	A	0.2	a	A	0.2	a	A	0.0	a	A	0.0	a	A

^(1)^ Comparison of variants; data flanked in each row by the same small letters (a, b, etc.) are not statistically different according to least significant differences test (*p* = 0.05) (n = 5). ^(2)^ Comparison of months; data flanked in each column by the same capital letters (A, B, etc.) are not statistically different according to least significant differences test (*p* = 0.05) (n = 5).

## Data Availability

Not applicable.
